# Insular Cortex—Biology and Its Role in Psychiatric Disorders: A Narrative Review

**DOI:** 10.3390/brainsci15080793

**Published:** 2025-07-25

**Authors:** Darko Laketić, Nikola M. Stojanović, Isidora Laketić, Milorad Pavlović, Bojan Milosević, Ana Starčević, Slobodan Kapor

**Affiliations:** 1 Faculty of Medicine, Niko Miljanić Institute of Anatomy, University of Belgrade, 11000 Belgrade, Serbia; drdarkolaketic@gmail.com (D.L.); ana.starcevic22@gmail.com (A.S.); kaporbg@gmail.com (S.K.); 2 Center for Mental Health Protection, University Clinical Center, 18000 Nis, Serbia; 3 IQVIA RDS, 11070 Belgrade, Serbia; laketicisidora28@gmail.com; 4 Clinic of Thoracic Surgery, University Clinical Center, 18000 Nis, Serbia; misapavlovicnis@yahoo.com; 5 Department of Surgery, Faculty of Medical Sciences, University of Kragujevac, 34000 Kragujevac, Serbia; drbojanzm@gmail.com

**Keywords:** insula, schizophrenia, bipolar disorder, anxiety disorders, gambling

## Abstract

The insular cortex has emerged as a key region implicated in a wide array of cognitive, emotional, and sensory processes. The anterior part of the insula (AIC) is central to emotional awareness, decision-making, and interoception, while the posterior insula (PIC) is more associated with somatosensory processing. The insula acts as a functional hub within the salience network and integrates homeostatic, affective, and cognitive information; thus, its role in different mental disorders seems to be prominent. Altered structure and connectivity of the insular cortex are evident in several psychiatric conditions. In schizophrenia, reductions in insular volume—especially on the left—correlate with hallucinations, emotional dysregulation, and cognitive deficits. Bipolar and major depressive disorders exhibit AIC volume loss and aberrant connectivity patterns linked to impaired affect regulation and interoceptive awareness. Anxiety disorders show functional hyperactivity of the insula, especially in response to fear-inducing stimuli, though findings on structural changes are mixed. Overall, growing evidence underscores the insular cortex’s central role in psychiatric pathophysiology and highlights its potential as a target for future diagnostic and therapeutic strategies.

## 1. Introduction

The insular cortex, or insula, was named by J.C. Reil back in the 19th century, who claimed that this structure is the seat of mental processes [1]. This structure is also referred to as “the fifth lobe of the brain”, “intralobular gyri”, and “intersylvian convolutions” [2]. In recent years, there has been growing interest in the insular cortex’s structure and function, especially in patients suffering from different mental disorders. Despite this, there is little information about this cortical region, partially owing to its location and to the fact that isolated insular region lesions are quite rare. A vast amount of information regarding the insular cortex’s structure and connectivity comes from studies on macaque monkeys. Some viewpoints suggest that the human insular cortex underlies mental capacities highly specific to humans. This work will describe the anatomy and physiology of the insular cortex and will try to associate the changes in the specific insular regions with different mental health disorders.

## 2. Anatomy, Histology, Neurophysiology, and Functional Connectivity of the Insular Cortex

**Figure 1:**
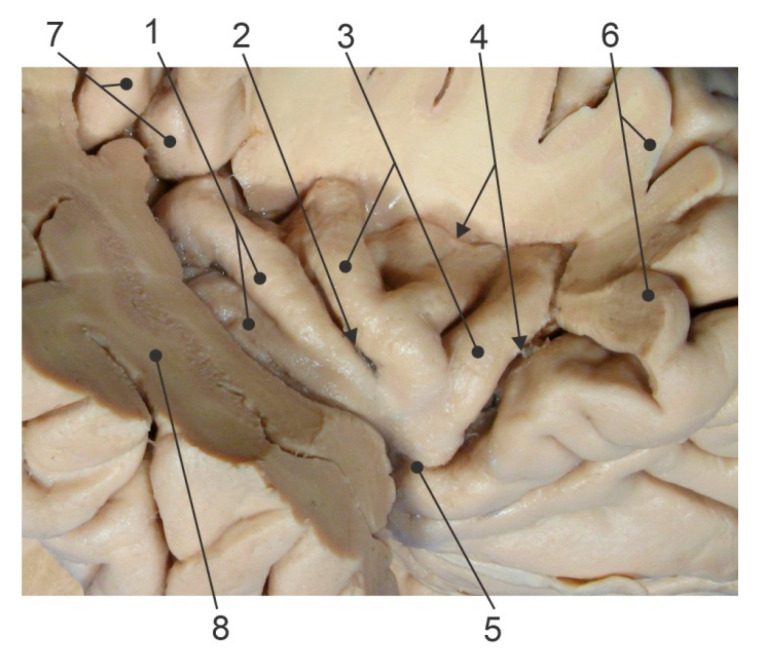
Anatomical features of insular cortex (side view, some cortical areas covering insula were removed); 1—posterior insular cortex (PIC) with anterior (ALG) and posterior (PLG) long insular gyrus marked by upper and lower full circle, respectively; 2—central insular sulcus (arrow); 3—anterior insular cortex (AIC), 4—circular insular sulcus; 5—limen insulae; 6—frontal operculum (removed); 7—parietal operculum (removed); and 8—temporal operculum (removed).

Anatomically, the insula is divided into anterior (AIC) and posterior (PIC) sections by a central insular sulcus (Figure 1). Morphologically, the anterior portion is divided by the anterior and precentral insular sulcus into the anterior (ASG), middle (MSG), and posterior (PSG) short insular gyrus. At the same time, the posterior portion is separated by the postcentral insular sulcus into the anterior (ALG) and posterior (PLG) long insular gyrus [3]. There are some other gyri that are often undeveloped or are missing and represent anatomical variations of this brain region.

The cytoarchitecture of the PIC has been investigated, while the AIC is still being studied. Based on the granularity of the IV layer, the insula can be separated into three subareas. The dorsal part of the PIC, which would correspond to the PLG, is a granular area, while the ventral part is a dysgranular (agranular) area [3], and the middle insula is disorganized [4]. The granular layer, a part of the neocortex, is distinguished from the agranular (allocortex) by the presence of an external granular layer (II) and internal granular layer (IV), and by the differentiation of the external pyramidal layer (III). The frontal part of the insula, as well as the anterior cingulate cortex (ACC), have characteristic neurons, Von Economo neurons (VENs), in the fifth layer. These largely unexplored spindle-shaped bipolar neurons are thought to play a role in intuition, complex social cognition, self-awareness, and decision-making, and are selectively susceptible to various neuropsychiatric and neurodegenerative disorders [5].

The most well-studied and partially explained neurotransmission at the level of the insula involves cholinergic transmission, with inputs arriving from the nucleus basalis of Meynert [6]. The insular cortex is rich in acetylcholine esterase [7], and neurons express alpha-4 and beta-2 nicotinic acetylcholine receptors [8]. It is believed, based on the action of physostigmine, a reversible acetylcholinesterase inhibitor, that cholinergic neurotransmission in this area is involved in the modulation of affect processing [9].

Based on the functional division of the insular cortex, it is suggested to have three distinct functional parts. The posterior insula is involved in the processing of afferent bodily information, the dorsal AIC (dAIC) in cognitive control (higher-order executive control), and the ventral AIC (vAIC) in emotional regulation and reward-based processes [10]. The processes underlying the mentioned structures could be independent, making this part of the cortex specialized, while at the same time, they can mutually coordinate, thus making the insular cortex integrative [3]. This potential explains why the insula serves as a network hub for numerous cognitive processes.

The insular cortex continues to be a ‘gating system’, allowing for the interplay between different neurocognitive processes [10]. Its connections have been initially established in animals where the frontal cortex, olfactory cortex, parietal lobe, cingulate cortex, somatosensory cortices, and the temporal lobe are connected with the insula. It is revealed that the AIC is more connected to the frontal and olfactory cortex, while the PIC has more connections to the cingulate and parietal cortex [11]. Later on, human studies found almost identical connectivity, with more precise connection patterns, demonstrating that the AIC has connections with the anterior cingulate, frontal, orbitofrontal, and anterior temporal areas, while the PIC primarily has connections with posterior temporal, parietal, and sensorimotor areas [3]. These connections allow us to associate the AIC with the emotional and language-related functions, and the PIC with somatosensory and auditory processing functions [12]. Described functional connectivity further reveals that the dAIC is connected to frontal, anterior cingulate, and parietal areas, vAIC with the limbic region, while the middle insula is connected with sensorimotor areas [3].

Afferent information from the parabrachial nucleus reaches insular cortices through the basal ventral medial nucleus (VMb) of the thalamus, thus providing descending control of the brainstem from which the impulse has initially risen [13]. The impulses arriving at the insular cortex are termed homeostatic impulses (modalities), which, in parallel, activate sensation and motivation, i.e., these feelings constitute emotions reflecting the survival needs of the body. Some of the impulses that could activate the insula include noxious stimuli (pain), temperature, itching sensations, muscle exercise, cardiorespiratory activation, hunger, thirst, and sensual touch [14]. The sum of inputs, including the ones from the ACC and interoceptive cortex, finally arrives at the right AIC that is suggested to be the location of the subjective awareness of the ‘feeling self’ [13]. Some authors found cardiac representation in the insular cortex [15]. After left insular cortex stimulation, the authors observed bradycardia or depression in heart activity, with the PIC having more impact than the AIC. On the contrary, right insular cortex stimulation more frequently evokes tachycardia [15].

The importance of the insula in the salience network has been one of the most studied roles of this brain region. The salience network involves several brain structures, which, apart from the insular cortex, include the ACC, amygdala, etc. Their role is to identify and separate the most homostatically relevant stimulus coming from a number of stimuli, either from the internal or external environment [16].

Also, the role of the insula in decision-making (especially risky decisions) has been recognized, since the internal cues from the nuclei located in the brain stem, activated with the disturbance in the internal milieu, trigger the insular cortex [17]. Connections between the insular cortex and other cortical regions, such as the pre-supplementary motor area and secondary somatosensory cortex, correlate with task performance and are activated during awarding stimuli [18]. The direct infusion of D1 receptor agonists in rats produces impulsive behavior [19], suggesting that the insular cortex might be associated with the control of impulsive behavior via dopaminergic neurotransmission.

## 3. Changes in Insular Cortex Structure and Function in Different Psychiatric Disorders

### 3.1. Schizophrenia

Schizophrenia (SCH) is a chronic and severe mental disorder characterized by distortions in thinking, perception, emotions, language, sense of self, and behavior. Common symptoms include hallucinations, delusions, disorganized thinking, and a reduction in social and emotional engagement [20,21]. There are several explanations for the genesis of SCH, and some of them are trying to find the connections between the changes in different brain regions and SCH symptoms’ intensity and disease progression.

From the developmental standpoint, the insular part of the brain cortex, together with the straight gyrus and the cingulate gyrus, are the earliest recognizable gyri at the stage of 16–18 weeks of gestation [22]. The fact that the changes in the insular cortex are observed in drug-naïve patients during the first psychotic episode points to the fact that this brain region is important in the pathophysiological process of SCH. By using MRI in drug-naïve patients with a first psychotic episode, significant morphological changes in the left insular cortical surface area were noted, which include a decrease in the cortical surface and gray matter volume [22]. Apart from the reduction in the cortical surface in first-psychotic-episode patients, the same has been observed in those with chronic SCH as well [12]. Also, subjects at risk of psychosis share morphological features of the insula with those suffering from SCH, which is indicative of the vulnerability of this brain area [23]. To further strengthen this theory, authors followed the changes in left insular cortex volume in subjects in an at-risk mental state, revealing that the volume reduction does not indicate further disease progression [24]. Interestingly, the right-sided insular volume has been rather indicative of patients who develop psychosis [24]. Additionally, a reduced and disorganized insular cortex has been postulated to have a disorganized connectome [25]. Also, it has been suggested that the changes in different cortical areas do not follow identical global decline, but rather follow a specific pattern [23].

Insula dysfunction in discriminating between self-generated and external information may contribute to the genesis of different types of hallucinations, which are a cardinal feature of SCH [26]. The severity of hallucinations strongly correlates with the reduction in the insular gray mass [27]. Apart from a global decrease in gray mass volume, it is found that the left insular gray mass volume is more pronounced than the right one [26]. There are studies suggesting that the right hemisphere superior temporal gyrus and insula gray matter have increased volume and greater activation, theoretically compensatory due to hypofunction of the left hemisphere, which might explain the genesis of acoustic hallucinations [28,29]. On the contrary, patients with somatic hallucinations have bilateral insular activation, and left middle frontal gyrus, right inferior frontal, and posterior cingulate gyrus activation, have also been observed [30].

Since the insular cortex is part of the limbic system, a decrease in blood flow through this region [31] suggests abnormal interaction within the limbic system and between the structures of the limbic system, and thus partially explains the emotional dysregulation seen in schizophrenia [31]. Adding to the notion of altered blood flow, an MRI study on connectomes showed intensified connections between the vAIC and superior temporal sulcus to be positively correlated with negative symptoms [25] such as anhedonia and diminished social interactions. As a part of the emotional blunting seen in SCH, there is a decreased spontaneous generation of vocal prosody, which has been connected with AIC dysfunction [26].

Also, one of the key symptoms in SCH is the disturbance in cognitive functioning, which is associated with different areas of the PFC [32]. A recent study revealed an increased functional connectivity between the insular cortex and various regions of the PFC, including the dorsolateral PFC (DLPFC), which is believed to be compensatory due to the hypofunction of DLPFC [33]. An observed reduction in the left insular gray mass volume, associated with negative symptomatology such as alogia and autism [34], can in part support the cognitive decline that has been associated with this brain region for a long time [23,31]. The connection between the AIC and the hippocampus has been found in patients with SCH to be more pronounced than in healthy subjects [33]. Since the hippocampus is essential for memory formation, and its activation is altered during memory formation in patients with SCH [35], it is suggested that this connection has a role in altered memory function in these patients.

### 3.2. Affective Disorders

Bipolar disorder (BD) is a chronic and incapacitating mental illness marked by alternating episodes of mania and depression, with depressive phases often lasting longer, occurring more frequently, and contributing heavily to psychosocial dysfunction and increased suicide risk [36]. BD and SCH share considerable overlap in genetic predisposition, brain structure and function, and clinical presentation, blurring traditional diagnostic boundaries such as the Kraepelinian dichotomy and complicating early identification, treatment, and management [33]. Although some clinical characteristics and biological markers have been proposed to differentiate BD from major depressive disorder (MDD), none have been incorporated into the standard diagnostic criteria, leaving the presence of mania as the only definitive marker, often absent or unclear in early stages [37].

Recent meta-analyses have found a bilateral decrease in AIC volumes in patients with BD [38,39], emphasizing the importance of this structure. The first meta-analysis found a reduction in the gray mass volume of the insular cortex in patients with BD, which has been associated with the altered emotional salience seen in these patients [38]. The results of the second meta-analysis, which encompassed 21 publications with more than 550 patients, showed a marked reduction in insular gray matter, which has been suggested to influence the clinical picture in BD patients [39] and draw the same conclusions as the first analysis [38]. As mentioned, the insula is an important part of a person’s emotional life and is involved in decision-making, which are both altered in BD patients. Furthermore, there are suggestions that the impaired insular function of internal awareness might be associated with an inadequate decision-making process seen during a manic episode [39]. In a recent analysis, a significant increase in connectivity between the vAIC and pACC has been discovered in BD patients, which can be explained by the gray matter abnormality and a decrease in its volume of the pACC [40]. Compared to the control group, there is an increased connection between the vAIC and dAIC and the thalamus and hippocampus in patients with BD [33]. The role of the thalamus, a relay nucleus in the forebrain circuits, which further include cortical structures [41], might be partially affected in BD patients by the changes in the connectivity with the AIC. The regulation of thalamic function can therefore be partially modulated by AIC activity and produce aberrant changes in BD patients’ emotional life, as well as in cognitive and social functioning [42]. There are also studies showing increased connectivity (hyperconnectivity) between mPFC and the insula, which has been suggested to play a role in poor executive control and increased attention to internal, emotionally salient processing in BD patients [43]. Furthermore, the accentuated hippocampal connection with the AIC [33] represents a common feature seen in BD and SCH patients, and might be connected with altered memory impairment. Other shared features between BD and SHC might involve an increase in activity between the insula and frontal cortex pole, describing some altered emotional aspects and cognitive dysfunction [33].

One of the most prominent symptoms seen in MDD patients is numerous somatic disturbances, and often an altered body awareness, which is believed to be associated with the insular cortex [44]. Some studies even suggest an altered, diminished perception of heartbeats in MDD patients, potentially associating it with interoceptive awareness, which is bottom line based in some theories stating that mood disorders are basically disorders of interoception [45]. In patients with MDD, there has been a reduction in the left insular gray mass volume, as well as a reduction in some other specific brain regions [46]. Decreased activation of the IC and other regions acting in emotion control have been seen in unmedicated patients with MDD, suggesting that this might be connected with the observed symptoms [47]. To further corroborate the involvement of the insula in MDD, studies showed increased functional connectivity between this region and the mPFC and ventromedial PFC, as well as increased regional metabolic activity in these patients [48,49]. Interestingly, there is also some association between insular dysfunction and food intake, both hyperphagia and hypophagia, in patients with MDD [50]; however, this requires further study. Finally, there is a strong biological background for vagal nerve stimulation in patients with MDD, which is based on the understanding of the connections and activation of the insular cortex and some other subcortical regions through vagal nerve carrying afferents from the periphery [45].

### 3.3. Anxiety Disorders

Anxiety disorders encompass several distinct mental conditions, which include generalized anxiety disorder (GAD), panic disorder (PD), specific phobia (SP), social anxiety disorder (SAD), agoraphobia (AP), and post-traumatic stress disorder (PTSD). Anxiety represents a specific, inborn, emotional, and cognitive reaction that enables humans to cope with everyday situations [51]. One needs to distinguish the emotion termed anxiety from fear, which might at first glance appear identical [52]. Although all drugs used in humans are screened for their efficacy in animals and pass through rigorous trials, it is evident that there are differences between anxiety in animals and humans. A recent animal study found that in the AIC, the agranular part, lesions have a crucial role in fear and anxiety regulation in mice [53], suggesting that this structure might play a role in human pathology as well.

The activation of insula, and its connections with the amygdala, hypothalamus, and periaqueductal gray matter, is involved in the processing of a number of negative emotions [54]. The role of the AIC as a part of the salience network, together with the amygdala and other cortical structures, should be taken into consideration when interpreting anxiety disorders, with a component of altered interoceptive representation. The alteration in insular processing and the modulation of emotions, both from outer and inner surroundings, can be expected in a variety of anxiety disorders. Also, bearing in mind the roles of the insula, which are associated with interoception, it is not unexpected that anxiety at both clinical and subclinical levels is associated with increased interoceptive accuracy [45]. Functional studies found that in radiolabeled flumazenil, a GABA_A_-benzodiazepine antagonist, binding is significantly decreased in the right insular cortex in patients with PD [55], suggesting the hyperactivity of this area. The insular hyperactivity, although to a different extent, has been observed in patients with SAD, SP, and PTSD, and is believed to be associated with the processing of negative feelings, such as disgust or anger [56]. Furthermore, the hyperactivation of this brain region in patients with SAD, SP, and PTSD follows the hypoactivation of cortical areas such as the ACC [56].

Early functional studies revealed that exposure to a phobic stimulus causes a bilateral increase in insular activity in patients with anxiety [57]. Also, the application of specific panic-provoking stimulants (lactate and cholecystokinin) led to an increased insular blood flow [58], associating this region with PD. An increase in blood flow through the insular region has been observed in patients with PTSD subjected to an imagery symptom provocation paradigm [59]. Similar alterations in blood flow through insular regions have been observed in patients with SAD [60] and SP [61].

From the morphometric point of view, there are some discrepancies in the view of insula volume changes that follow anxiety disorders. A reduction in insula volume [62] and cortical thickness [63] has been observed in patients with SAD, while at the same time, some early studies show an increased insular volume [64]. Also, a reduction in insular volume has been observed in patients with GAD [65] and PD [66].

### 3.4. Gambling Disorder

Gambling is often perceived as a recreational activity, which can escalate into a serious behavioral addiction known as gambling disorder. This disorder is characterized by a persistent and recurrent pattern of problematic gambling behavior, causing significant distress and impairment in personal, financial, and social functioning. These patients struggle to control their impulses and continue gambling despite negative consequences affecting all aspects of their lives. The condition shares similarities with substance use disorders, and it often co-occurs with other mental health conditions such as depression, anxiety, and substance abuse [67].

Morphological studies found that the insular cortex volume has been increased in subjects suffering from internet gambling [68]. Some foundations of the roles of the insular cortex in gambling disorder are also laid down by studies examining its connections with other brain regions. Thus, the connections between the PIC and supplementary motor area, or some other area associated with sensorimotor processes, are activated during monetary-based decision-making [69]. Furthermore, the dorsal part of the AIC is connected with the dorsolateral PFC, which is associated with higher cognition and executive control processes [70]. At the same time, the ventral AIC is directly connected to the ventral striatum and the orbitofrontal cortex, which are deemed to be a reward system [70]. From this information, it is suggested that the ventral AIC associates the impulses from the reward system with the ones coming from the other mentioned regions.

The results of a recent study indicate a positive connection between the ventral AIC, putamen, cerebellum, occipital, temporal, precentral, and central operculum regions, and at the same time, some negative connections with the orbitofrontal cortex [10]. Also, insular lesions have been found to interfere with performance in the test mimicking gambling. With these and previously mentioned facts in mind, it is somewhat hard to completely grasp the brain dynamics associated with gambling, since it is hard to directly pinpoint either hypoactive or hyperactive brain regions. However, it is quite obvious that underlying certain addiction-related behaviors is altered insular connectivity with different brain regions, driving away from goal-oriented processes towards addiction-related ones [10].

To better follow the changes in structure and function, as well as the association of these changes with symptoms of different mental health disorders, a summary of the findings previously discussed has been given in Table 1. The table illustrates some of the most meaningful changes discussed in the manuscript, and helps summarize all findings on the changes in insular cortex volume and connectivity. The most obvious overlap is between SCH and BD, which indeed share some biological background, with a decrease or alteration in insular connectivity with other brain regions. On the opposite spectrum are anxiety disorders, where the increased connectivity of this region with other brain structures is highlighted.

## 4. Future Perspectives

Since there are some implications that the early development of the insular cortex might contribute to neurodevelopmental risk in psychiatric disorders, longitudinal imaging studies in at-risk populations could reveal critical windows for intervention and identify early structural changes. These investigations would reveal if prenatal stress, maternal inflammation, or early-life adversity are responsible for disease occurrence and progression, bearing in mind that the insula consists of distinct subregions with specific functions and connectivity patterns, and that some future research might be focused on mapping these subdivisions in detail and analyzing how their network disruptions contribute to symptoms associated with mental health disorders.

In this review, several mental health disorders have been reviewed, including schizophrenia, bipolar disorder, major depression, and anxiety disorders, and the insular cortex is found to be affected across all of them, which makes this structure a potential transdiagnostic biomarker. Future research should adopt dimensional frameworks to examine how insular dysfunction relates to core behavioral dimensions like emotion regulation, interception, and cognitive control. This shift from categorical to dimensional models could lead to the development of insula-based biomarkers for early detection, prognosis, and treatment stratification across a spectrum of mental illnesses.

One should approach the insular cortex as a target for several mental disorders, which can be achieved indirectly via modulation with transcranial magnetic stimulation (TMS) or transcranial direct current stimulation (tDCS). Also, vagal nerve stimulation (VNS) and deep brain stimulation (DBS), targeting insula-connected circuits, may modulate symptoms in treatment-resistant depression and anxiety. These approaches hold potential promise due to the complex connectivity of the insular cortex with limbic and prefrontal areas. Thus, future studies should investigate the efficacy of such approaches using personalized targeting strategies based on individual connectome profiles. Real-time fMRI neurofeedback could also serve as a novel tool to train individuals in regulating insular activity consciously.

Bearing in mind that the insular cortex is central to interoception, which is increasingly recognized as disrupted in mood, anxiety, and somatic disorders, some future research should explore how the cortex integrates visceral, immune, and hormonal signals via vagal afferents, particularly in relation to the gut–brain axis. This includes the role of microbiota, gut inflammation, and autonomic regulation in shaping insular activity. Disorders such as irritable bowel syndrome, functional somatic syndromes, and eating disorders may especially benefit from this approach. A better understanding of these pathways could foster novel treatments, including dietary interventions, vagal stimulation, or microbiota-targeted therapies for interoceptive dysfunction.

## 5. Conclusions

Although there is some understanding of the anatomical and connectional changes in the insular cortex of patients suffering from different psychiatric disorders, some questions remain unanswered. One such question is regarding the extent of the overlap between different mental disorders that potentially share identical changes in the insular cortex. Other questions are related to the extent of the changes observed in different stages of the mental illness. Finally, one needs to wonder whether the changes in the insular cortex are related to disease progression or whether they represent the foundation of the disorder. Future studies should focus on determining the exact neural pathways that are associated with a specific mental disorder(s). Through this, we could potentially target, e.g., medically or through transcranial magnetic stimulation, the structure(s) associated with the disease/symptom, and thus alleviate them. Additionally, future research should adopt longitudinal and cross-diagnostic designs to determine whether insular changes precede, accompany, or result from clinical symptoms. Mapping insular subregions and their functional networks using high-resolution neuroimaging, combined with artificial intelligence and multimodal integration, could uncover transdiagnostic biomarkers and guide personalized treatments. Finally, a better understanding of the insula’s role in interoception and its interaction with peripheral systems, such as the gut–brain axis, may offer novel insights into both the pathophysiology and treatment of psychiatric disorders.

## Figures and Tables

**Table 1:** Summarized changes in the insular cortex in different psychiatric disorders.

Disorder	Insular Cortex			
	Area	Volume Change	Functional Change	Potential Symptom Association
Schizophrenia	Left insular cortex	Volume reduction	Disrupted connectivity with temporal, frontal, and limbic areas	Hallucinations, emotional dysregulation, cognitive deficits, and memory disturbances
Bipolar Disorder	Bilateral AIC	Volume reduction	Increased AIC connectivity with ACC, thalamus, and hippocampus	Emotional dysregulation and cognitive deficits
Major Depressive Disorder	Left insular cortex	Volume reduction	Decreased activation; increased functional connectivity with mPFC and vmPFC	Interoceptive dysfunction, emotional blunting, and food intake regulation
Anxiety Disorders	Volume reduction in SAD, GAD, and PD	Hyperactivity in the right insula in PD, SAD, SP, and PTSD	Increased connectivity with the amygdala, hypothalamus, and periaqueductal gray, coupled with ACC hypoactivation	Altered interoception and emotional salience processing
Gambling Disorder	NS	Increased volume	Altered connectivity with motor, cognitive, and reward-related regions	Affects decision-making

ACC—anterior cingulate cortex; AIC—anterior insular cortex; GAD—generalized anxiety disorder; mPFC—medial prefrontal cortex; NS—no specific area; PD—panic disorder; PTSD—post traumatic stress disorder; SAD—social anxiety disorder; vmPFC—ventromedial prefrontal cortex.

## Abbreviations

AIC	Anterior Insular Cortex
ACC	Anterior Cingulate Cortex
AP	Agoraphobia
BD	Bipolar Disorder
CSF	Cerebrospinal Fluid
DLPFC	Dorsolateral Prefrontal Cortex
GABA	Gamma-Aminobutyric Acid
GAD	Generalized Anxiety Disorder
IC	Insular Cortex
MDD	Major Depressive Disorder
MRI	Magnetic Resonance Imaging
mPFC	Medial Prefrontal Cortex
PD	Panic Disorder
PFC	Prefrontal Cortex
PIC	Posterior Insular Cortex
pACC	Pre-Genual Anterior Cingulate Cortex
PTSD	Post-Traumatic Stress Disorder
SAD	Social Anxiety Disorder
SCH	Schizophrenia
SP	Specific Phobia
vAIC	Ventral Anterior Insular Cortex
vmPFC	Ventromedial Prefrontal Cortex
VNS	Vagal Nerve Stimulation
